# Suitable concentrations of brassinolide application enhances growth performance and saikosaponin biosynthesis by transcriptional activation of triterpenoid pathway genes in *Bupleurum chinense* DC

**DOI:** 10.3389/fpls.2025.1517434

**Published:** 2025-03-31

**Authors:** Shengwei Zhou, Linlin Yang, Jie Wan, Lu Chen, Xupeng Gu, Lu Qiao, Leixia Chu, Ning Dong, Chengming Dong, Weisheng Feng

**Affiliations:** ^1^ School of Pharmacy, Henan University of Chinese Medicine, Zhengzhou, China; ^2^ Henan Provincial Ecological Planting Engineering Technology Research Center of Daodi Herbs, Zhengzhou, China; ^3^ Co-construction Collaborative Innovation Centre for Chinese Medicine and Respiratory Diseases by Henan & Education Ministry of PR China, Henan University of Chinese Medicine, Zhengzhou, China; ^4^ The Engineering and Technology Center for Chinese Medicine Development of Henan Province, Henan University of Chinese Medicine, Zhengzhou, China; ^5^ Department of Stomatology, The First Affiliated Hospital of Henan University of Chinese Medicine, Zhengzhou, China

**Keywords:** *Bupleurum chinense* DC., brassinolides, saikosaponin, quantitative real-time PCR, terpenoid-targeted metabolomics, secondary metabolism synthesis

## Abstract

The role of brassinolides (BRs) in regulating the synthesis of plant secondary metabolites has been recognized. However, the effect of brassinolides on the synthesis of saikosaponin in *Bupleurum chinense* DC. (*B. chinense*) is still unresolved, To address this knowledge gap, experiments were conducted in which different concentrations (0 mg/L as CK, 0.1 mg/L, 0.2 mg/L, and 0.4 mg/L) of BRs solution were sprayed on *B. chinense* taproot in the present study. We measured the growth indicators of each group of *B. chinense*, used quantitative real-time PCR (qRT-PCR) to determine the expression level of genes related to the biosynthesis of saikosaponin, used terpenoid-targeted metabolomics to determine the accumulation of saikosaponin, and verified the metabolomics results by HPLC. Following a 12-day treatment with the 0.2 mg/L BRs solution, the fresh and dry root weights, the taproot length, and the taproot diameter of *B. chinense* escalated by 60.35%, 60.11%, 25.17%, and 28.07% respectively, in comparison with the CK group. The expression of genes related to the biosynthesis of saikosaponin (*HMGR, DXR, IPPI, FPS, SE, P450-2*, and *P450-3*) significantly increased. Moreover, a terpenoid-targeted metabolomic investigation identified 27 distinct saikosaponins, inclusive of saikosaponin A and D, with a notable accumulation observed in 17 saikosaponins. The HPLC findings indicated that the contents of saikosaponin A and D elevated by 72.64% and 80.75% respectively when treated with 0.2 mg/L BRs solution. Conversely, the treatment of 0.4 mg/L BRs solution did not exhibit any significant alteration in the concentrations of saikosaponin A and D when compared to the CK group. In conclusion, the 0.2 mg/L BRs solution demonstrates a more pronounced regulatory impact on the synthesis of saikosaponin A and D. Our investigation revealed that the accumulation of these crucial medicinal bioactive compounds, saikosaponin A and D, can be enhanced through the application of a 0.2 mg/L BRs solution in the ecological cultivation of *B. chinense*.

## 1 Introduction

*Bupleurum chinense* DC. (*B*. *chinense*) is a perennial plant of the Umbelliferae family (Lin et al., 2008). The 2 020 edition of the *Pharmacopoeia of People’s Republic of China* that its roots can be used as medicine (Tian et al., 2009). In the Eastern Han Dynasty 2 000 years ago, Zhang Zhongjing’s book *Treatise on Febrile and Miscellaneous Disease* first recorded this traditional Chinese medicine, which has the effects of invigorating qi, relieving depression, clearing away heat and expelling evil (Chen et al., 2020; Sun et al., 2024). In China, *B*. *chinense* is mostly distributed in Henan, Shanxi, Shanxi, Shandong, Gansu, Jilin provinces and other places. Among them, the best is in Song County, Henan, and it is also known as “Songhu”. Several traditional Chinese medicine prescriptions use *B*. *chinense*, such as *Xiao Chaihu Decoction* and *Qingfei Paidu Decoction*, which are effective against the coronavirus (COVID-19) (Lyu et al., 2021). In China, the annual demand for *B. chinense* is 10,000-15,000 tons, but the annual supply is only about 8,000 tons. This makes *B. chinense* in short supply and prices rise, currently standing at approximately 100 yuan per kilogram, equivalent to roughly $14 per kilogram. The secondary metabolites in *B*. *chinense* are mainly triterpenoid saikosaponin (SS-s), such as saikosaponin A (SS-a) and D (SS-d). Saikosaponin A and D are both pentacyclic triterpenoids and are optical isomers of each other (Zhang et al., 2015). They are composed of triterpenoid aglycone and its carbon skeleton is composed of six isoprene units. The sugar moiety is β-D-glucopyranosyl-(1→3)-β-D-fucopyranosyl (Jia et al., 2022). The contents of saikosaponin A (SS-a) and saikosaponin D (SS-d) is between 0.29% and 1.52% (Yang et al., 2022a), which is low, so increasing the content of saikosaponin is conducive to the improvement of *B*. *chinense*’s quality.

**Figure 1:**
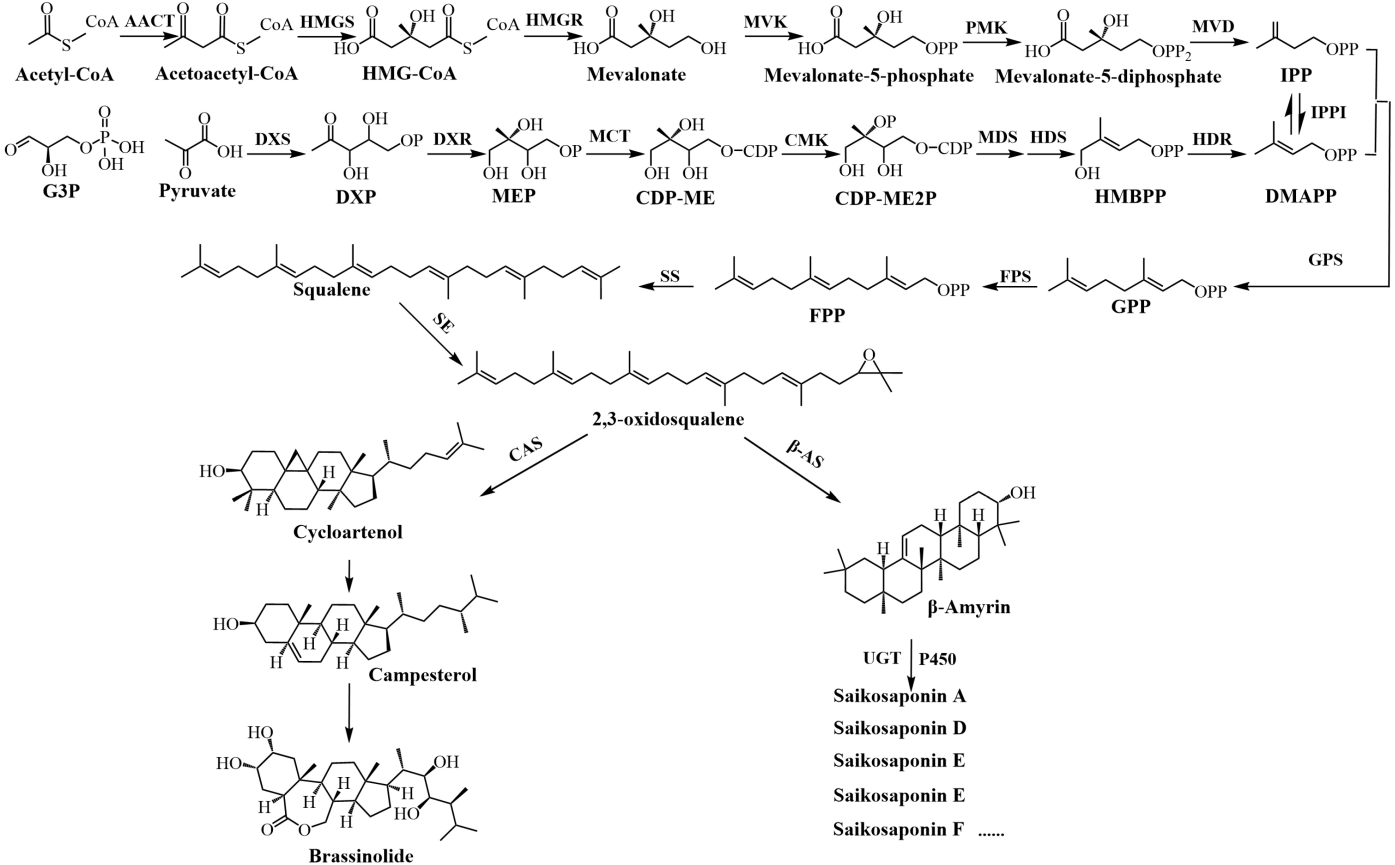
Synthetic pathway of saikosaponin and brassinolide.

Saikosaponins(SS-s) are oleanane-type triterpenoid saponins (Wang et al., 2021). Saikosaponin such as saikosaponin A and D have a series of pharmacological effects such as anti-inflammatory and anti-depressant. SS-a activates tet1/dll3/notch1 signalling and promotes hippocampal neurogenesis to improve depression-like behavior in mice (Tong et al., 2024). SS-d (8 mg/kg) improves intestinal inflammation in mice with ulcerative colitis (UC) induced by dextran sulfate sodium (DSS) by inhibiting NF-kappa B activation and regulating intestinal flora (Li et al., 2020). The biogenesis of triterpenoid saponins is facilitated through two distinct synthetic routes: the cytoplasmic mevalonate (MVA) pathway and the plastid 2-C-methyl-D-erythritol-4-phosphate (MEP) pathway (Thimmappa et al., 2014). **Figure 1** shows the synthesis pathway of saikosaponin. The mevalonate (MVA) pathway, beginning with acetyl-CoA as the primary substrate, synthesises of Acetyl-CoA by acetyl-CoA C-acetyltransferase (AACT), creates isopentenyl pyrophosphate (IPP) via a sequence of five condensation reactions. Alternatively, the 2-C-methyl-D-erythritol-4-phosphate (MEP) pathway utilizes pyruvate and glyceraldehyde 3-phosphate(G3P) as initial substrates, synthesises of MEP by DXP synthetase (DXS) and reductoisomerase (DXR)subsequently produces dimethylallyl pyrophosphate (DMAPP) through a series of seven reaction steps (Zhao and Li, 2018). Among them, IPP and DMAPP are the precursors of the parent nucleoside of triterpenoid saponins, and the MVA pathway plays a leading role in the biosynthesis of triterpenoid saponins. HMG-CoA reductase (HMGR) is the first rate-limiting enzyme in the MVA pathway (Choi et al., 1992), catalyzing the conversion of HMG-CoA to mevalonate. This reaction is an irreversible process and is also an important regulatory point in the biosynthesis of terpenes. IPP and DMAPP are condensed under the action of GPP synthetase (GPS) to form geranyl pyrophosphate (GPP); GPP is then catalyzed by FPP synthetase (FPS) to add a second IPP unit to form farnesyl pyrophosphate (FPP); then the two FPPs are condensed by squalene synthase (SS) to form squalene, which is then epoxidized by squalene epoxidase (SE) to form 2,3-oxidosqualene (Zhao and Li, 2018). Among them, FPS, SS, and SE are all located at the branch points of the triterpenoid saponin synthesis pathway and are key enzymes involved in extending the triterpenoid saponin parent nucleoside skeleton. β-Amyrin was synthesized from 2,3-oxidosqualene by β-amyrin synthase (β-AS), and various saikosaponins were synthesized by cytochrome P450 (P450) and glycosyltransferase (UGT) (Mizutani and Ohta, 2010; Seki et al., 2015).

Research has established that 2,3-oxidosqualene serves as a shared substrate for the production of BRs and saikosaponin. This substrate is ultimately converted into saikosaponins via the β-AS pathway, or transformed into cycloartenol through the cycloartenol synthase (CAS) pathway, culminating in the synthesis of brassinolide (Zhao and Li, 2012). BRs are a group of polyhydroxy phytosteroid hormones that are essential for many aspects of plant life (Nolan et al., 2020). BRs have the ability to stimulate plant growth, enhance plant physiological metabolism, and consequently augment plant yield and quality (Ali, 2017). It has been reported to increase the yield and quality of crops or fruits such as grape (*Vitis vinifera* L. cv. Alphonse Lavallée) (Babalık et al., 2020) and maize (*Zea mays* L. cv. Zhengdan958) (Gao et al., 2017). BRs also have a significant effect on the regulation of plant secondary metabolites (Li et al., 2016). Spraying treatment with 0.5 mg/L BRs solution increased the total phenol content of peppermint (*Mentha piperita* L.) by 2 times compared with the CK group (Çoban and Göktürk Baydar, 2016); In the case of tea (*Camellia sinensis* (L.) O. Ktze.), 0.1 mg/L solution of BRs markedly amplified the expression of *CsHMGR* and substantively elevated the concentration of tea polyphenols, catechins, amino acids, and caffeine (Wen et al., 2024), and non-targeted metabolomics results showed that differential metabolites were mainly enriched in the caffeine synthesis pathway. Nonetheless, there is a paucity of research investigating the mechanism through which BRs regulates the synthesis of saikosaponin in *B. chinense*.

We based on these recent reports and the role of BRs in the secondary metabolism of various plant species, we hypothesized that BRs may affect the content of saikosaponin and thus the quality of *B*. *chinense*. To elucidate the specific effects of BRs on its growth and quality, we administered varying concentrations of BRs – 0 mg/L (CK), 0.1 mg/L, 0.2 mg/L, and 0.4 mg/L - to the root systems of *B. chinense* for the purpose of assessing their impact on key growth parameters such as taproot length, stem length, taproot diameter, and weights of dry root, fresh root, and above-ground parts. Additionally, we evaluated the relative expression levels of pivotal enzymatic genes (*AACT*, *HMGR*, *DXS*, *DXR*, *IPPI*, *FPS*, *SS*, *SE*, *β-AS*, *P450-1*, *P450-2*, *P450-3*, *P450-7*, *UGT8* and *UGT7382*). We executed a systematic analysis and comparison of the influence of a 0.2 mg/L BRs solution on diverse metabolites within the *B. chinense* taproots via targeted metabolomics. The effect of BRs on the quality of *B. chinense* was examined by quantitatively measuring the contents of saikosaponins A, D, C, E and F. Our results showed that BRs significantly promoted the growth and quality of *B. chinense*.

## 2 Materials and methods

### 2.1 Experimental materials and processing methods

*B. chinense* seedlings were collected from the standardized planting base of medicinal plants in Song County, Luoyang City, Henan Province, China (34°7′30″N, 112°3′35″E). Healthy, disease-free, and robust seedlings about 1 year old were transplanted into planting pots. The flower pot specifications are 10cm in diameter at the top, 8cm in diameter at the bottom, and 20cm in height. There are holes at the bottom of the flower pots. Each pot has one seedling and is filled with nutrient soil. All *B. chinense* seedlings were placed in the greenhouse of Henan University of Traditional Chinese Medicine (34°46′N, 113°4′8E) with good ventilation. All potted plants were watered appropriately to keep the soil moisture content at 27-32% for 30 days. One month later, the *B. chinense* seedlings grew to 18 cm in height and entered a vigorous growth state, and were then treated and regulated with BRs solution.

**Figure 2:**
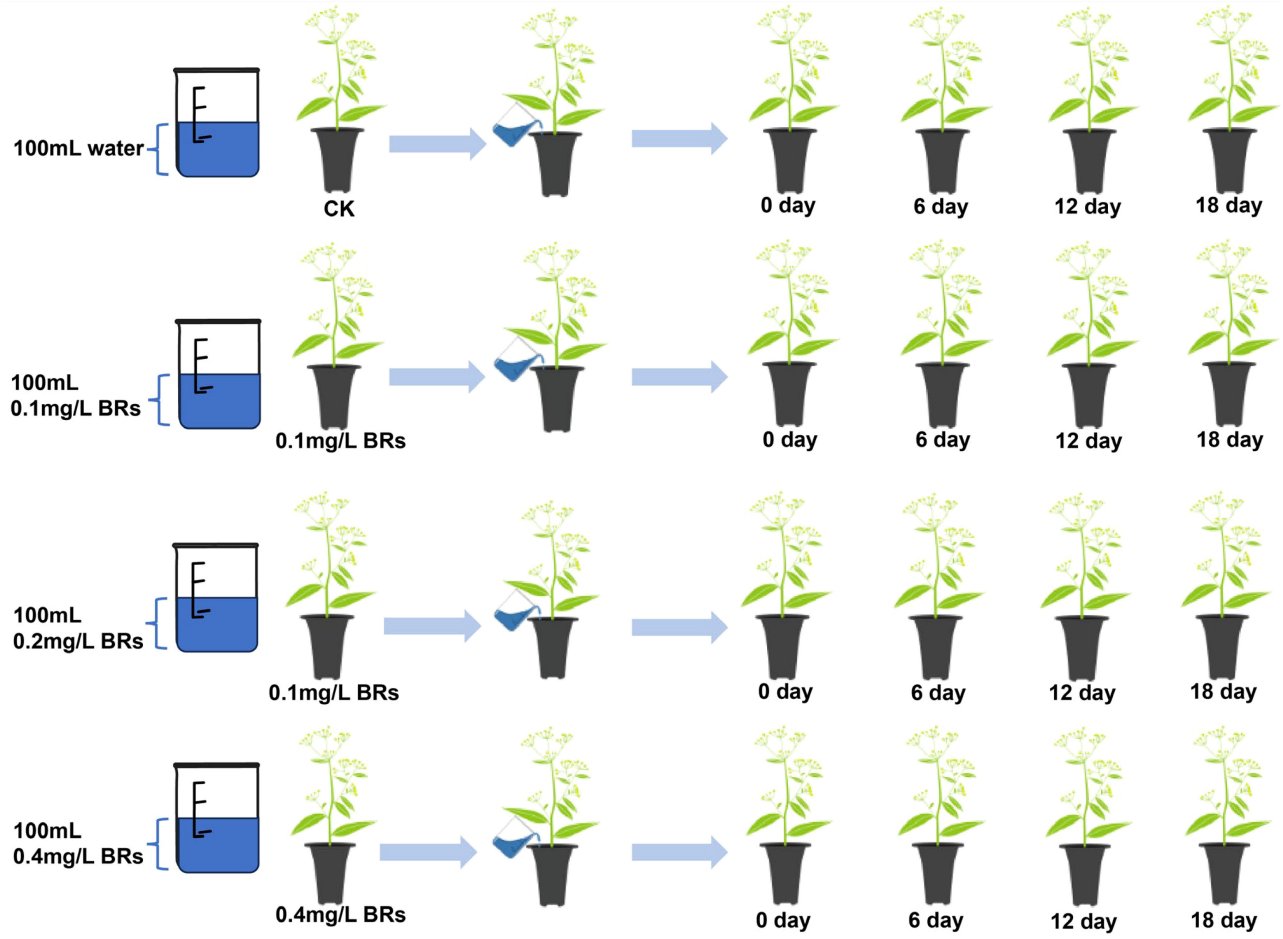
Sample processing for this experiment.

A total of 144 *B. chinense* seedlings, exhibiting uniform growth, were segregated into four distinct groups, each containing 36 seedlings. *B. chinense* roots were then subjected to irrigation with 0mg/L BRs (CK), 0.1mg/L BRs, 0.2mg/L BRs, and 0.4mg/L BRs solution respectively, each administered once every three days in 100mL quantities, until the completion of the final sampling. Fresh root samples were obtained post-treatment at intervals of 0, 6, 12, and 18 days (**Figure 2**). These samples were subsequently cleaned, with some of them instantly frozen in liquid nitrogen and stored in an ultra low-temperature freezer at -80°C. The remaining samples were dried and pulverized in anticipation of subsequent determination of pertinent indices.

### 2.2 Determination of *B. chinense* growth indexes

The taproot and stem length of *B. chinense* were quantified employing a steel ruler, while the root diameter was assessed using an electronic vernier caliper. The mass of the fresh and dry roots, along with the stem and leaf, were evaluated using a high-precision balance (XPR303S/AC, Mettler-Toledo International Inc, Switzerland), accurate to 1/1000th of a gram.

### 2.3 Determination of key genes in the biosynthesis pathway of saikosaponins

Total RNA was isolated from plant samples using the New Plant RNA Kit For Polysaccharides & Polyphenolics-Rich (ZP434, Beijing Zoman Biotechnology Co., Ltd.). This isolated total RNA was then employed as a template, and subsequently reversed transcribed into cDNA using the HiScript III RT SuperMix (R323-01, Nanjing Vazyme Biotech Co., Ltd.), which was preserved at -20°C. Utilizing EF-1α as the endogenous reference gene, the relative gene expression levels of key enzymes involved in saikosaponin biosynthesis pathway (*AACT*, *HMGR*, *DXS*, *DXR*, *IPPI*, *FPS*, *SS*, *SE*, *β-AS*, *P450-1*, *P450-2*, P450-3, *P450-7*, *UGT8* and *UGT7382*) in the roots of *B. chinense* were RT-qPCR. The reaction system refers to the study of Yang et al. (2019). Reaction system: PowerTrack™ SYBR Green Master Mix, 10uL; forward primer, 1uL; reverse primer, 1uL; cDNA, 1uL; ddH_2_O, 7uL. Primers information is shown in **Supplementary Table S3**.

### 2.4 Targeted metabolomics analysis of *B. chinense*

The treatment methods of metabolomics refer to the studies that have been reported (Qu et al., 2022), The method has been slightly modified. Metabolite extraction: Samples from the 0.2 mg/L BRs group and CK group (n = 3), kept at -80°C, were subjected to vacuum freeze-drying using a lyophilizer (Scientz-100F). They were then ground to a powder (30 Hz, 1.5 min) using a MM400 grinder (Retsch). Subsequently, an electronic balance (MS105DM) was used to measure 50 mg of the sample powder, to which 1200 μL of pre-cooled 70% methanolic water internal standard extract was added at 20°C (using less than 50 mg for every 1200 μL of extractant per 50 mg of sample). Vortex mixing was performed every 30 minutes for 30 seconds, a total of 6 times. Afterward, the mixtures were centrifuged (rotational speed 12,000 rpm, for 3 minutes), and the supernatant was collected. The samples were then filtered through 0.22 μm pore-sized membranes and stored in injection vials for further analysis with UPLC-MS/MS.

Ultra-performance liquid chromatography (UPLC)-MS/MS system: The extract of *B. chinense* sample was analyzed by ultra-high performance liquid chromatography-electrospray tandem mass spectrometry system (UPLC, ExionLC™ AD, https://sciex.com.cn/) and tandem mass spectrometry system (https://sciex.com.cn/). The analysis conditions were as follows: UPLC: chromatographic column, Agilent SB-C18 (1.8 µm, 2.1 mm * 100 mm); the mobile phase was solvent A, pure water containing 0.1% formic acid, and solvent B, acetonitrile containing 0.1% formic acid. The sample was determined using a gradient program with the starting conditions of 95% A and 5% B. A linear gradient was programmed to 5% A, 95% B within 9 min, and the composition of 5% A, 95% B was maintained for 1 min. Subsequently, it was adjusted to a composition of 95% A, 5.0% B within 1.1 min and maintained for 2.9 min. The flow rate was set to 0.35 mL/min; the column temperature was set to 40°C; and the injection volume was 2 μL. The effluent was alternately connected to an ESI-triple quadrupole linear ion trap (QTRAP)-MS. The ESI source operating parameters were: source temperature 550°C; ion spray voltage (IS) 5500 V (positive ion mode)/-4500 V (negative ion mode); ion source gas I (GSI), gas II (GSII), and curtain gas (CUR) were set to 50, 60, and 25 psi, respectively; collision activated dissociation (CAD) was high. QQQ scans were acquired as MRM experiments, and the collision gas (nitrogen) was set to medium. DP (declustering potential) and CE (collision energy) were performed on each MRM transition, and DP and CE were further optimized. A specific set of MRM transitions were monitored in each period based on the metabolites eluting in that period.

Bioinformatic analysis of targeted metabolomics datasets: The metabolites identified were annotated via the KEGG compound database, after which the metabolites that had been annotated were mapped onto the KEGG pathway database. The metabolomics data were analyzed using the open-source software, metaX, which facilitated both univariate and multivariate analyses to discern the metabolites that varied in accumulation between the 0.2 mg/L BRs group and the CK group. The employed methodologies included both parametric and non-parametric tests, differential expression multivariate analysis, principal component analysis (PCA), and orthogonal partial least squares discriminant analysis (OPLS-DA). To prevent overfitting, a permutation test with 200 permutations was executed. Metabolites were considered differentially accumulated if the three conditions were simultaneously fulfilled: (1) a fold change ≥ 1.2 or ≤ 0.833; (2) a Wilcoxon test P-value ≤ 0.05; (3) a variable importance in projection (VIP) score ≥ 1.

### 2.5 Determination of the contents of five saikosaponins by HPLC

The treatment methods of HPLC refer to the studies that have been reported (Yang et al., 2019). Preparation of sample solution: The sample preparation process begins with the grinding of dried *B. chinense* roots that have undergone a 12-day treatment period, followed by sieving through a No. 3 sieve to procure the requisite fine powder. The powder (0.4g) is then weighed and placed into a stoppered conical flask, followed by the addition of 10mL ammonia methanol solution (1:9, v/v), with the flask’s weight taken post-addition. The flask is then shaken for 30 seconds, followed by a 40-minute ultrasonication at 35°C in an ultrasonic bath with specifications of 500W power and a 50KHz frequency. The flask is cooled post-ultrasonication, reweighed, and any weight deficit countered with additional ammonia methanol solution (1:9, v/v). The sample solution is then filtered, yielding the filtrate. This extraction process is repeated thrice, with all filtrates combined in an evaporating dish. The combined filtrates are then evaporated in a 50°C water bath, reconstituted with methanol, and adjusted to the required volume in a 10mL volumetric flask. After thorough agitation, the sample is filtered through a 0.22 μm membrane filter, making it ready for HPLC assessment.

The binary gradient elution system consists of acetonitrile (A) and water (B), and the following gradient program is used to achieve separation: 0-5 min, 0-30% A; 5-10 min, 30-35% A; 10-20 min, 35-50% A; 20-25 min, 50-52% A. The injection volume was 20 μL, the flow rate was 0.7 mL/min, and the system operating temperature was 20°C. The detection wavelength was 200 nm.

The saikosaponin standards used in this experiment were all from Shanghai yuanye Bio-Technology Co., Ltd. A methanol solution encompassing saikosaponins A (LOT: D13IB235166), D (LOT: D13IB235166), C (LOT: DO11GB163264), E (LOT: MG28GB150097), and F (LOT: A07IB211849) is prepared and diluted to the necessary concentration to construct a calibration curve, incorporating seven concentrations of the five compounds. A quasi-curve, reflective of the concentration alterations of each analyte, is formed utilizing the peak areas, as illustrated in **Supplementary Figure S1**. The standard regression equations for saikosaponins A, D, C, E, and F are respectively given as Y = 0.0635X + 0.9892 (R^2^ = 0.9991), Y = 0.0399X + 0.9314 (R^2^ = 0.9995), Y = 0.0735X + 1.2658 (R^2^ = 0.9996), Y = 0.016X + 0.2175 (R^2^ = 0.9994), and Y = 0.076X + 1.3692 (R^2^ = 0.9993). By substituting the peak areas of the five saikosaponins ascertained via high-performance liquid chromatography (HPLC) into the standard regression equations, the concentrations of the five saikosaponins are obtained. Consequently, the content of the saikosaponins in each 0.4 g sample can be calculated by multiplying the concentrations by the volume of the sample solution (10 mL).

### 2.6 Statistical analysis

The ultimate empirical data were subjected to statistical analysis utilizing SPSS software (version 19.0, SPSS Inc., USA). Bioinformatics scrutiny was executed using the Metware Cloud, a complimentary online platform for data examination (https://cloud.metware.cn). To enhance the visual representation of the experimental outcomes, graphical illustrations were created using GraphPad software (version 10.0, GraphPad Software, Inc., USA).

## 3 Results

### 3.1 Analysis results of growth indicators of *B. chinense*

**Figure 3:**
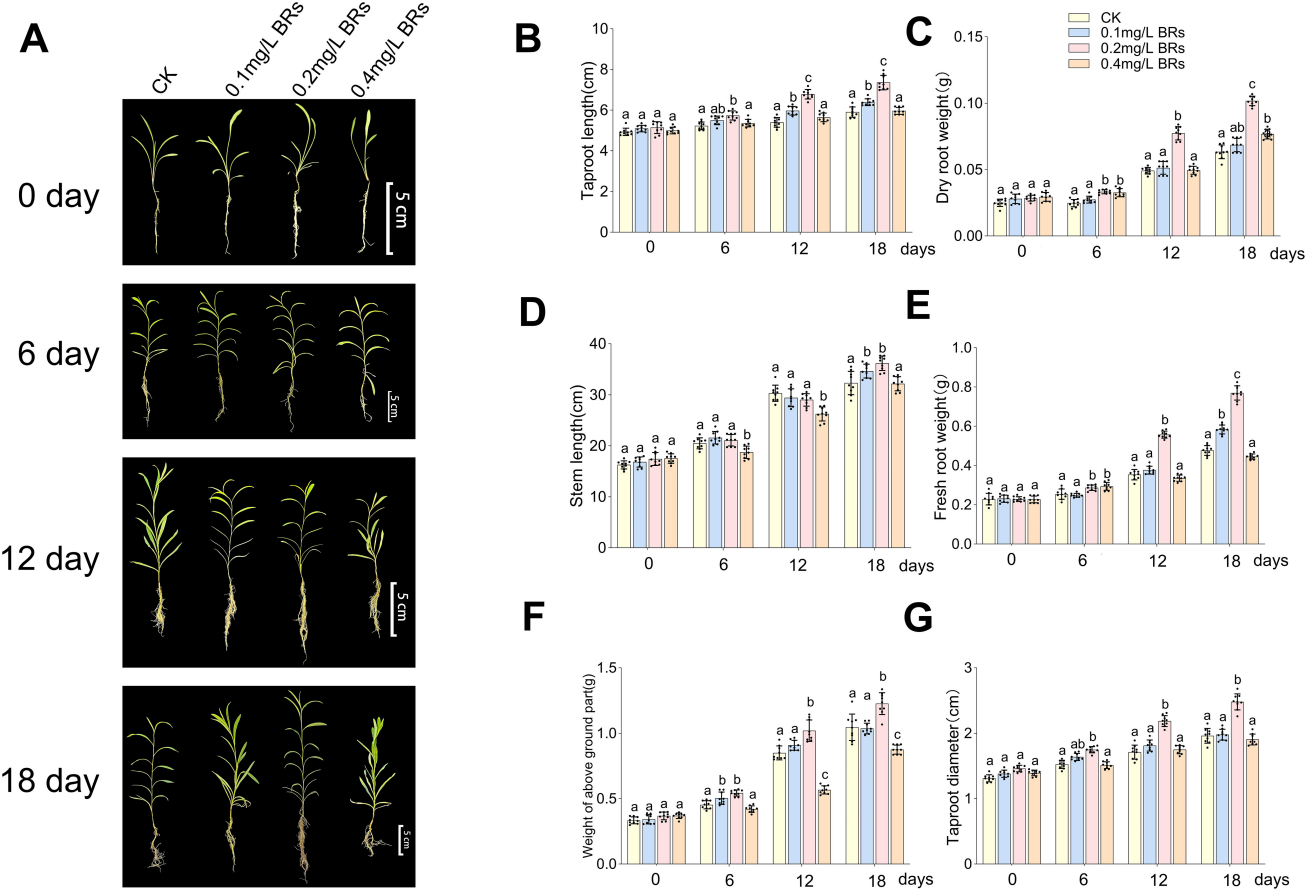
The data of changes in various growth indicators of *B. chinense* under 0mg/L BRs (CK), 0.1mg/L BRs, 0.2mg/L BRs, and 0.4mg/L BRs solution are expressed as mean ± SDs (standard deviation, n = 9). **(A)** shows the growth of *B. chinense* at each sampling time. **(B–G)** shows the taproot length **(B)**, dry root weight **(C)**, stem length **(D)**, fresh root weight **(E)**, weight of above ground part **(F)**, and taproot diameter **(G)** of *B. chinense*. Duncan’s one-way ANOVA was used, and different lowercase letters indicate significant differences among treatments (P< 0.05).

**Figure 3** illustrates the variations in several growth parameters of *B. chinense*. After six days of treatment, no significant change was discerned across all indicators. However, with the prolonged treatment period, significant differences emerged across the treatments of 0mg/L BRs (CK), 0.1mg/L BRs, 0.2mg/L BRs, and 0.4mg/L BRs solution on the 12th day (**Figure 3A**). The taproot lengths of *B. chinense* (**Figure 3B**) were respectively measured as 5.40 ± 0.21cm, 5.97 ± 0.29cm, 6.78 ± 0.21cm, and 5.64 ± 0.29cm. The dry root weights were 0.05 ± 0.002g, 0.05 ± 0.004g, 0.08 ± 0.004g, and 0.05 ± 0.003g, respectively (**Figure 3C**). The stem lengths (**Figure 3D**) were respectively 31.08 ± 1.40cm, 29.42 ± 1.57cm, 29.00 ± 1.09cm, and 26.25 ± 1.23cm. The fresh root weights (**Figure 3E**) were recorded as 0.35 ± 0.024g, 0.38 ± 0.018g, 0.56 ± 0.016g, and 0.34 ± 0.014g, respectively. The weights of the above-ground section (**Figure 3F**) were 0.85 ± 0.048g, 0.91 ± 0.034g, 1.02 ± 0.074g, and 0.57 ± 0.028g, respectively. The diameters of the taproot (**Figure 3G**) were recorded as 1.71 ± 0.097mm, 1.81 ± 0.075mm, 2.19 ± 0.075mm, and 1.75 ± 0.055mm, respectively. Except for the stem length, all other indices reached their peak values under the 0.2 mg/L BRs solution, demonstrating a significant deviation from the control (*P < 0.001*), and this significant divergence was retained on the 18th day (*P < 0.001*). This suggests that a 0.2 mg/L BRs solution promotes the growth and development of *B. chinense*.

### 3.2 Expression levels of genes involved in saikosaponin biosynthesis

**Figure 4:**
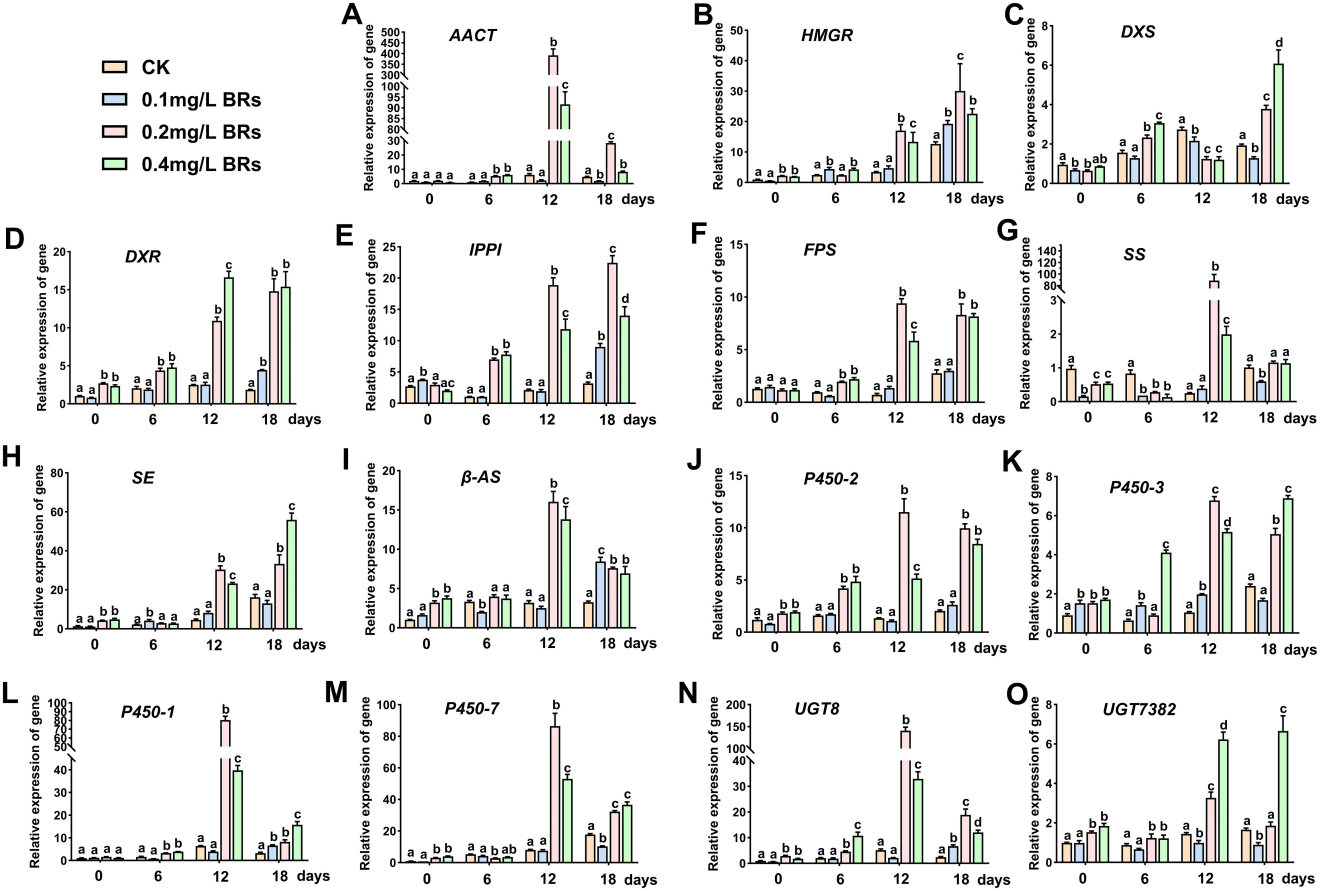
Effects of 0.1mg/L BRs (CK), 0.1 mg/L BRs, 0.2 mg/L BRs, and 0.4 mg/L BRs solution on the expression levels of genes related to the saikosaponin biosynthesis pathway. **(A–O)** show the relative expression of *AACT* **(A)**, *HMGR* **(B)**, *DXS* **(C)**, *DXR* **(D)**, *IPPI* **(E)**, *FPS* **(F)**, *SS* **(G)**, *SE* **(H)**, *β-AS* **(I)**, *P450-2* **(J)**, *P450-3* **(K)**, *P450-1* **(L)**, *P450-7* **(M)**, *UGT8* **(N)**, and *UGT7382* **(O)**, respectively. Data are expressed as mean ± SDs (standard deviation, n = 3). Duncan’s one-way ANOVA was used, and different lowercase letters indicate significant differences among treatments (*P*< 0.05).

The molecular mechanisms underlying the influence of various BRs concentrations, particularly the 0.2 mg/L BRs solution, on the synthesis and accumulation of saikosaponin were investigated, focusing on alterations in gene transcription levels. **Figure 4** displays that, following a 12-day treatment with a 0.2 mg/L BRs solution, relative expression levels of *AACT*, *SS*, *β-AS*, *P450-1*, *P450-7* and *UGT8* were significantly elevated, with increases of 63.89, 352.44, 5.01, 12.52, 10.79 and 27.39 folds (*P < 0.05*) compared to the CK (**Figures 4A, G, I, L–N**). However, these levels significantly dropped after 18 days. The relative expression levels of *HMGR*, *DXR*, *IPPI*, *FPS*, *SE*, *P450-2* and *P450-3* significantly increased after 12 days of treatment with 0.2 and 0.4 mg/L BRs solution, maintaining this upward trend on the 18th day (**Figures 4B, D–F, H, J, K**). Specifically, their relative expression levels after a 0.2 mg/L BRs solution on the 12th day were 5.55, 4.38, 8.95, 13.06, 6.95, 8.89 and 6.65-fold (*P < 0.05*) the CK group’s levels, respectively. Following a 12-day treatment with 0.2 and 0.4 mg/L BRs solution, *DXS’s* relative expression significantly decreased to 46.1% and 44.5% (*P < 0.05*) of the CK (**Figure 4C**), respectively, then significantly increased on the 18th day, reaching 1.91 and 3.27-fold (*P < 0.05*) the CK’s levels, respectively. After treatment with 0.4 mg/L BRs solution, *UGT7382’s* relative expression significantly increased on both the 12th and 18th days, reaching 4.55-fold and 3.80-fold (*P < 0.05*) the control’s level, respectively (**Figure 4O**). Hence, we determined that, with the exception of *DXS* and *UGT7382*, the relative expression levels of other genes associated with saikosaponin synthesis were significantly upregulated compared to the control on the 12th day after treatment with 0.2 mg/L BRs solution (*P < 0.05*). Consequently, we selected the samples from the CK group and the 0.2 mg/L BRs group on the 12th day for metabolomics correlation analysis.

### 3.3 Targeted metabolomics analysis of *B. chinense*

**Figure 5:**
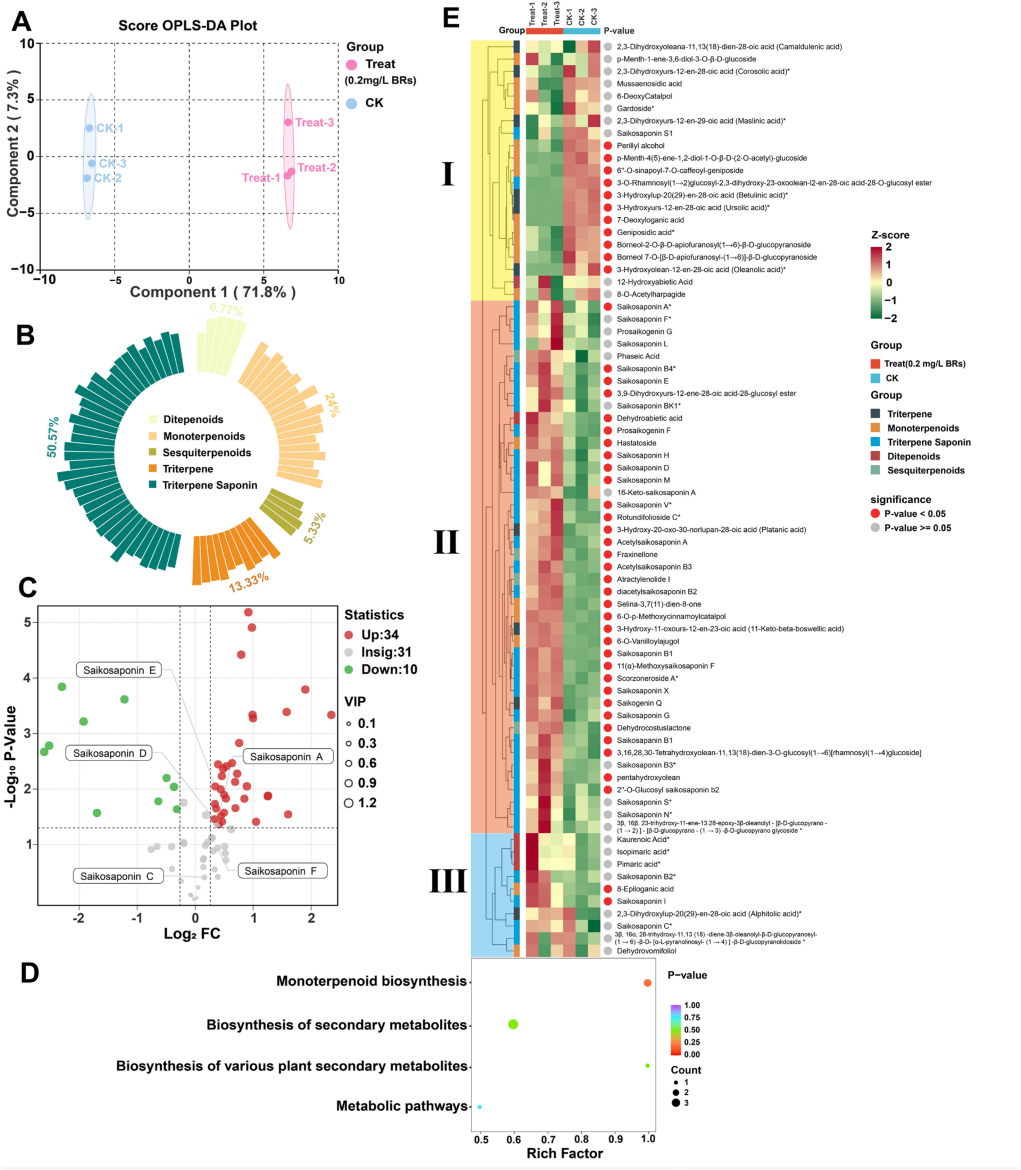
Basic metabolite information of 0.2mg/L BRs group and CK group based on terpene targeted metabolomics. **(A)** OPLS-DA scoring plot of metabolites. **(B)** Categories and proportions of metabolites. **(C)** Volcanoplot was used to analyze the significance of metabolites between the 0.2 mg/L BRs group and the CK group analysis. **(D)** Statistical analysis of metabolites enriched in the terpene biosynthetic pathway using KEGG annotation. **(E)** Terpenoid metabolite relative calorimetry plot.

To further reveal the differences in saikosaponin metabolites between the CK and 0.2 mg/L BRs group, we performed terpenoid-targeted metabolomics analysis on the 12th day samples. The basic metabolite information is shown in **Figure 5**. Orthogonal partial least squares discriminant analysis (OPLS-DA) analysis is a multivariate statistical method with supervised pattern recognition, which can effectively propose effects unrelated to the study and thus screen differential metabolites. In order to maximize the reflection of the differences between CK and 0.2 mg/L BRs group, OPLS-DA was used to establish a relationship model between terpenoid metabolite expression and sample category (**Figure 5A**). In this model, R^2^X and R^2^Y represent the explanation rate of the constructed model for the X and Y matrices, respectively, Q^2^ represents the predictive ability of the model, and Q^2^>0.9 indicates that the constructed model is very suitable (**Supplementary Figure S2**). The OPLS-DA score plot showed that the first and second principal components (Component1 and Component2) explained 71.8% and 7.3% of the total variance, respectively, and the explained amount of the total variance was significant, indicating that there were good differences in the sample types and metabolite expressions between CK and 0.2 mg/L BRs group. After identification and KEGG annotation, terpenoid metabolites were divided into different secondary metabolite categories, including Ditepenoids, Monoterpenoids, Sesquiterpenoids, Triterpene, and Triterpene Saponin (**Figure 5B**). In this study, a total of 75 terpenoid metabolites were detected, including 5 Ditepenoids, 18 Monoterpenoids, 4 Sesquiterpenoids, 10 Triterpene, and 38 Triterpene Saponin, accounting for 6.77%, 24%, 5.33%, 13.33%, and 50.57% of the total metabolites, respectively. To screen out differential terpene metabolites, the following screening conditions were met at the same time: (1) fold change ≥ 1.2 or ≤ 0.833; (2) Wilcoxon test P value ≤ 0.05; (3) metabolites with important variables in prediction (VIP) ≥ 1 were considered differential terpene metabolites. The results showed that there were 44 differential terpenoid metabolites in total, of which 34 terpenoid metabolites were significantly up-regulated and 10 terpenoid metabolites were significantly up-regulated. Among them, saikosaponin A, D and E in the treatment group was 1.30, 1.26, and 1.27 folds that of the CK group, respectively (**Figure 5C**). All differential terpenoid metabolites were matched to the KEGG database to obtain the pathway information involved in the terpenoid metabolites. The annotated results were enriched and analyzed to obtain the pathways with more differential terpenoid metabolites. The differential terpenoid metabolites were mainly annotated and enriched in the monoterpenoid biosynthesis pathway (**Figure 5D**). The monoterpenoid biosynthesis pathway synthesizes upstream substances, which are closely related to the synthesis of saikosaponins. The differences in metabolite accumulation patterns among different samples can be analyzed using cluster heat maps (**Figure 5E**).

**Figure 6:**
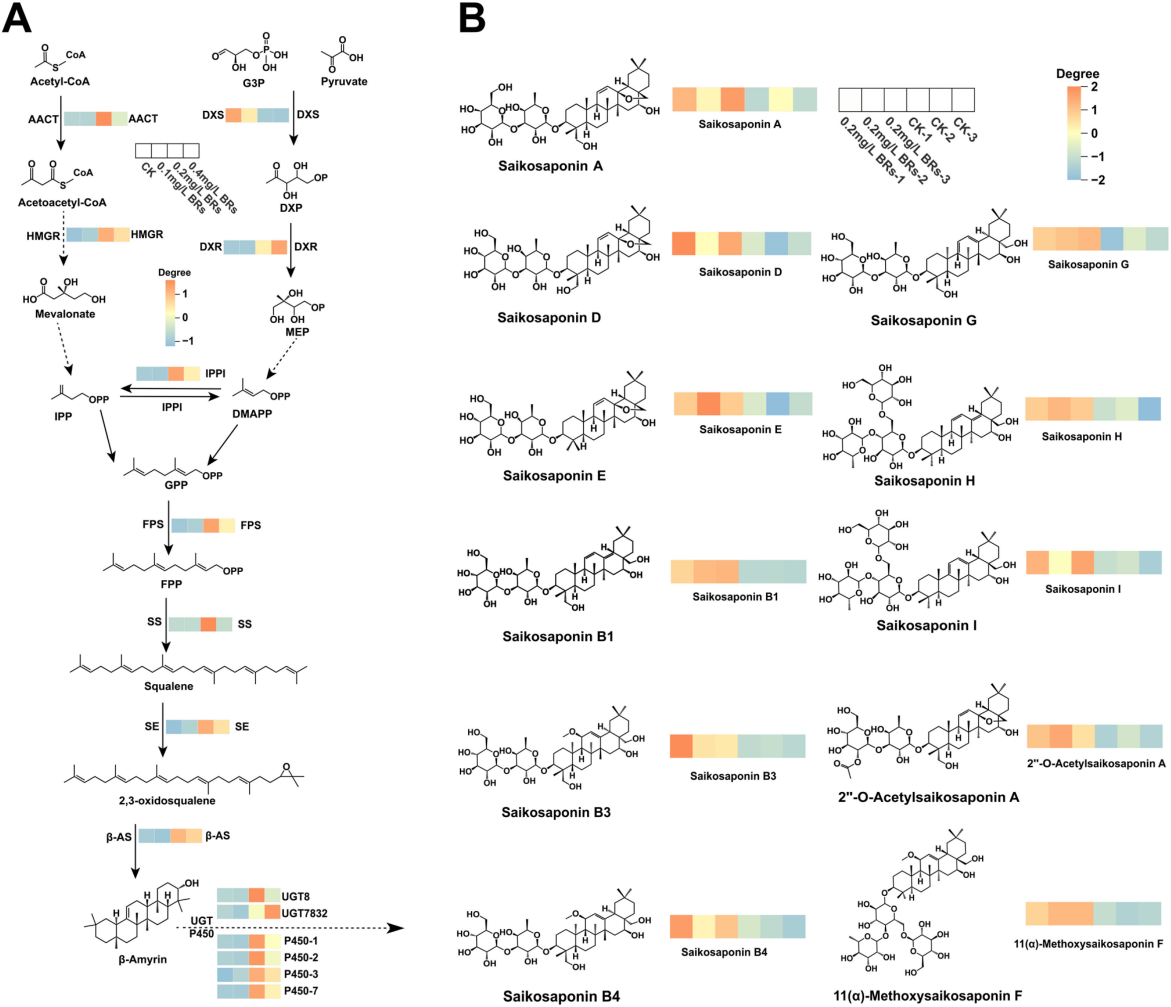
**(A)** Expression of key genes in the saikosaponin biosynthesis pathway in *B*. *chinense* after 12 days treatment. **(B)** Heat map of the relative amounts of triterpenoid saponins in *B*. *chinense*.

From **Figure 6A**, we can clearly see that the expression of saikosaponin synthesis key enzyme gene is significantly up-regulated in the 0.2 mg/L BRs group. Predominantly, triterpene saponin represented the majority of metabolites in cluster II. Following the application of a 0.2 mg/L BRs solution, the levels of 11 saikosaponin metabolites (Saikosaponin A, D, E, B1, B3, B4, G, H, I, 2’’-O-Acetylsaikosaponin A, and 11(α)-Methoxysaikosaponin F) exhibited a significant increase (**Figure 6B**). This observation aligns with the outcome that the relative expression of genes associated with the saikosaponin synthesis pathway was enhanced in the 0.2 mg/L BRs group.

### 3.4 Contents of five saikosaponins in *B. chinense*

**Figure 7:**
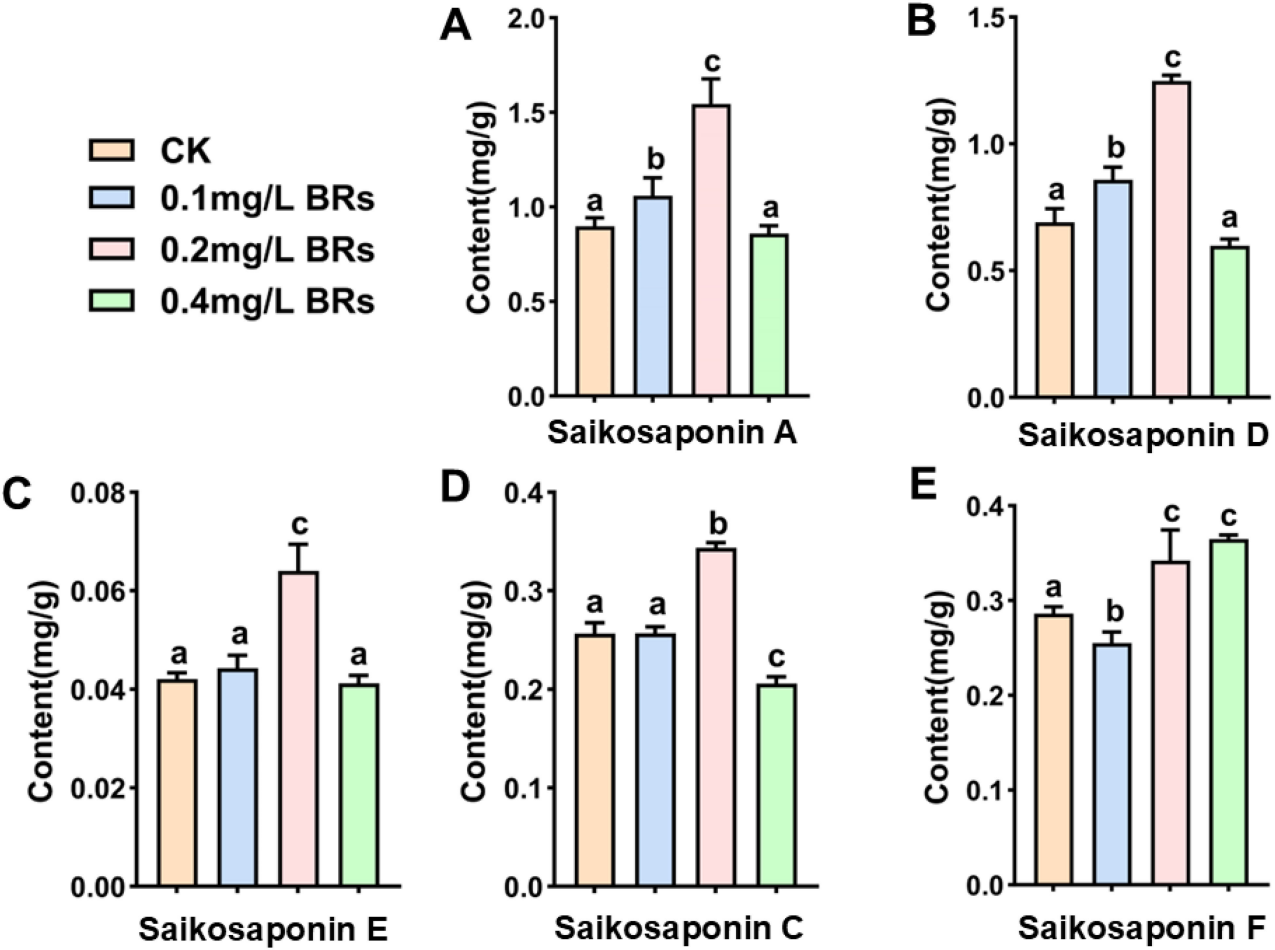
Effects of 0mg/L BRs (CK), 0.1 mg/L BRs, 0.2 mg/L BRs, and 0.4 mg/L BRs soluton on the content of saikosaponin. **(A–G)** shows the contents of saikosaponin **(A)**, saikosaponin **(D)**, saikosaponin **(E)**, saikosaponin **(C)**, and saikosaponin **(F)**, respectively. Data are expressed as mean ± SDs (standard deviation, n = 3). Duncan’s one-way ANOVA was used, and different lowercase letters indicate significant differences among treatments (*P*< 0.05).

An HPLC analysis was conducted to determine the content of these five triterpenoids in the roots of *B. chinense* following treatment with varying concentrations of BRs for a duration of 12 days (**Figure 7**). The content of these five triterpenoids, in decreasing order, is saikosaponin A, D, F, C and E. Saikosaponin A and D are key medicinal components in *B. chinense*. The content of saikosaponin A with the application of CK, 0.1 mg/L BRs, 0.2 mg/L BRs, and 0.4 mg/L BRs treatment were 0.914 ± 0.004 mg/g, 1.042 ± 0.012 mg/g, 1.578 ± 0.024 mg/g, and 0.865 ± 0.015 mg/g, respectively (**Figure 7A**), while the content of saikosaponin D were 0.691 ± 0.006 mg/g, 0.861 ± 0.016 mg/g, 1.249 ± 0.022 mg/g, and 0.598 ± 0.008 mg/g, respectively (**Figure 7B**). The content of saikosaponin A and D surged by 72.64% and 80.75%, while saikosaponin E (**Figure 7C**), saikosaponin C (**Figure 7D**), and saikosaponin F (**Figure 6E**) augmented by 52.38%, 33.85%, and 19.58% respectively, compared to the CK group (*P<0.05*). These findings were in accordance with the terpenoid targeted metabolomics analysis. Thus, it can be deduced that the 0.2 mg/L BRs solution significantly enhances the content of saikosaponin A, D, F, C, and E in *B. chinense*.

## 4 Discussion

Saikosaponin and BRs are both terpenoids, and both have common synthesis substrates and pathways (Yao et al., 2020). However, people still lack understanding of the mechanism by which BRs affect the synthesis of saikosaponin. This study quantitatively analyzed the effects of 0mg/L BRs (CK), 0.1 mg/L BRs, 0.2 mg/L BRs, and 0.4 mg/L BRs solution on the growth of *B. chinense*, the expression of related genes, terpenoid targeted metabolome analysis and the contents of saikosaponin A, D, C, E, and F. 0.2 mg/L BRs solution can promote the growth of *B. chinense* taproots, and at the same time, it can increase the expression of saikosaponin biosynthesis genes (*AACT*, *HMGR*, *DXR*, *IPPI*, *FPS*, *SS*, *SE*, *β-AS*, *P450-1*, *P450-2*, *P450-3*, *P450-7*, *UGT8*, *UGT7382*). Terpenoid targeted metabolomics showed that differential terpenoid metabolites were mainly annotated and enriched in the monoterpene biosynthesis pathway, which is closely related to the synthesis of upstream substances of saikosaponin. HPLC results showed that 0.2 mg/L BRs solution had a positive effect on the content of saikosaponin A, D, C, E and F, which also verified the results of qRT-PCR and terpenoid targeted metabolomics. This study clarified the mechanism of action of 0.2 mg/L BRs solution on the synthesis and accumulation of saikosaponin compounds in *B. chinense*, and provided a theoretical basis for the cultivation and production of *B. chinense*.

Brassinolides (BRs), a microscale yet potent endogenous plant hormone, was initially extracted from rapeseed pollen by American scientists in 1979 (Clouse, 2001). BRs influence the mechanical properties of cell walls, facilitating cell expansion driven by turgor pressure, promoting elongation of plant cells, and subsequently enhancing the growth of plant roots, stems, and leaves. This process improves photosynthesis, ultimately boosting crop yields (Clouse, 2002). Research by Maia et al. demonstrated that the application of BRs enhanced CO_2_ influx and fixation, amplified the absorption of water and nutrients by tomato roots, thereby augmenting tomato yields (Maia et al., 2018). BRs can also stimulate corn to enhance carbon source absorption, leading to increased yields (Gao et al., 2017). Aras et al. applied different concentrations of BRs to lavandin (*Lavandula x intermedia* Emeric ex Loisel.) and found that 1.5 mg/L BRs not only significantly promoted the its growth, but also increased the content of essential oils and total phenols (Asci et al., 2019). In this study, After a 12-day treatment with a 0.2mg/L BRs solution, the fresh root weight, dry root weight, taproot length, and taproot diameter of *B. chinense* exhibited increases of 60.35%, 60.11%, 25.17% and 28.07%, respectively, compared to the CK group (*P<0.05*). No significant variation was observed following treatment with 0.2mg/L BRs and 0.4mg/L BRs solution. This may be attributed to the fact that the 0.2 mg/L BRs solution facilitated the elongation and enlargement of *B. chinense* root cells, leading to increased taproot weight. Concentrations of BRs that are either too low or too high are not conducive to the growth and development of *B. chinense* taproot.

BRs influences the composition of secondary metabolites in plants by modulating the expression of genes responsible for the synthesis of these metabolites. An analysis of transcriptome data related to alkaloid synthesis in *Pinellia ternate* (Thunb.) Druce revealed that post-BRs treatment, the expression levels of alkaloid synthesis genes (*PAL*, *ANAS*, *CHY* and *4CL*) significantly escalated compared to the control group, thereby resulting in a 90.87% increase in the total alkaloid content (Guo et al., 2022). Numerous pivotal genes in the triterpenoid saponin biosynthesis pathway, including *HMGR*, *IPPI*, *FPS*, *SS*, *SE*, *β-AS*, *UGT* and *P450*, have been identified in *B. chinense* (Kim et al., 2006; Dong et al., 2011; Sui et al., 2021). However, the role of BRs in gene regulation within the triterpenoid saponin biosynthesis in *B. chinense* remains elusive. Our findings reveal significant differences in the regulation of related genes by various concentrations of BRs solution. The 0.1mg/L BRs solution exhibits a limited impact on gene expression within the triterpene saponin synthesis pathway. In contrast, the 0.2mg/L and 0.4mg/L BRs solution markedly enhance the expression of genes associated with triterpene saponin synthesis (*HMGR*, *DXR*, *IPPI*, *FPS*, *SE*, *P450-2* and *P450-3*) (*P < 0.05*), with the 0.2 mg/L BRs solution demonstrating a particularly notable positive effect. The 0.2mg/L BRs solution also significantly boosts the expression levels of *AACT*, *SS*, *β-AS*, *P450-1*, *P450-7* and *UGT8* (*P< 0.05*). Compared to the 0.4 mg/L BRs solution, the 0.2 mg/L BRs solution exhibits a more pronounced regulatory impact on the synthesis of saikosaponin A and D in *B. chinense*. The plant increases the content of saikosaponin A and D in its roots by upregulating the expression of saikosaponin biosynthesis genes, demonstrating superior adaptability to the 0.2 mg/L BRs solution.

Metabolomics, a subfield of systems biology (Yang et al., 2022b), performs simultaneous qualitative and quantitative analyses of all low-molecular-weight metabolites in an organism or cell at a specific physiological stage (Sampaio et al., 2016). The biosynthetic mechanism forms the crux of secondary metabolic studies in medicinal plants (Tripathi et al., 2016). The application of metabolomics technology to discern the relationship between exogenous hormones and the biosynthesis of secondary metabolites in medicinal plants, and to elucidate the regulatory mechanism of the secondary metabolite network, is vitally important for understanding the genesis of active ingredients and enhancing medicinal plant cultivation technology. When tea (*Camellia sinensis* (L.) O. Ktze.) were treated with 0.1 mg/L BRs solution, the expressions of *CsHMGR*, *CsGDH* and *CsGs* were significantly up-regulated. Meanwhile, 18 metabolites related to tea quality were detected by metabolomics, which were mainly enriched in the caffeine pathway (Wen et al., 2024). Metabolomic analysis showed that 5 mg/L brassinolide treatment increased the yield of *Opuntia ficus-indica* L. fruits and seeds, the content of phenols and flavonoids in the pulp, and the proportion of linoleic acid in the seeds (Atteya et al., 2022), In this research, terpenoid-targeted metabolomics was employed to dissect the alterations in metabolites in the roots of *B. chinense* when exposed to 0.2 mg/L BRs solution. The analysis revealed that the 0.2 mg/L BRs treatment significantly influenced the distribution and variety of terpene metabolites. For *B. chinense*, saikosaponin is the most critical metabolite and was thus the focal point of this research. The terpenoid-targeted metabolome analysis identified a total of 27 saikosaponins, and in conjunction with saikosaponin A and D, a cumulative of 17 saikosaponins significantly accumulated in response to the 0.2 mg/L BRs solution. This outcome also coincided with the findings of the HPLC and qRT-PCR) analysis. The terpenoid-targeted metabolomics unveiled the accumulation mechanism of saikosaponins in the roots of *B. chinense* under the influence of the 0.2 mg/L BRs solution and its adaptability to BRs regulation.

BRs can influence not only the growth and development of plants but also modulate the synthesis and accumulation of secondary metabolites. A treatment with 0.1 mg/L BRs significantly escalated the total alkaloid content in *Pinellia ternate* by 90.87% and the bulb yield by 29.67% (Guo et al., 2022). Similarly, a 0.1 mg/L BRs solution substantially elevated the carotenoid content in *Pinellia ternate* (Zhang et al., 2024). After administration of 0.5 ppm BRs solution for 24 h, the concentration of catechins and theanine increased, while the concentration of caffeine remained unchanged (Li et al., 2016). However, the effect of BRs on saikosaponin content in *B. chinense* has not been reported. In the current study, the levels of saikosaponin A and D in the 0.2 mg/L BRs solution were augmented by 72.64% and 80.75% respectively compared to the CK (*P < 0.05*). However, the saikosaponin A and D contents in the 0.1 mg/L BRs solution showed a relatively modest increase of 14.00% and 24.60% respectively (*P < 0.05*), indicating distinct optimal BRs concentration for diverse plants. Furthermore, no significant variance was noted in the saikosaponin A and D levels when treated with 0.4 mg/L BRs solution compared to the CK, suggesting that higher BRs concentrations are not beneficial for the accumulation of saikosaponin A and D. This could be attributed to the common synthetic substrate shared by saikosaponin and BRs. Applying high concentrations of BRs externally may amplify the BRs content in *B. chinense*, which may consequently reduce the synthesis of the common substrate, leading to a decreased accumulation of saikosaponin A and D.

In this study, by measuring the growth indices of *B. chinense*, the relative expression levels of key synthase genes for saikosaponin, terpenoid metabolomics, and the content of saikosaponin, it was discovered and confirmed from four perspectives that the 0.2 mg/L BRs solution, which can promote the accumulation of saikosaponin in *B. chinense*. This provides a theoretical basis and strategy for improving the planting technology and quality of *B. chinense.*

## 5 Conclusion

In the present investigation, we elucidated the metabolic mechanism of *B. chinense* under varying concentrations of BRs. Despite 0.1 mg/L BRs solution fostering the growth of *B. chinense*, it did not yield a positive impact on the accumulation of saikosaponins. Conversely, the 0.2 mg/L BRs solution enhanced the concentrations of saikosaponin A and D by upregulating the expression of saikosaponin biosynthesis genes. A terpene-targeted metabolome analysis further ratified the positive influence of 0.2 mg/L BRs solution on saikosaponin synthesis. The results of the above study suggest that the accumulation of saikosaponin A and D, which are crucial medicinal bioactive compounds, can be facilitated by utilizing a 0.2 mg/L BRs solution in the ecological cultivation of *B. chinense*. This may be helpful in improving the quality of *B. chinense*. Of course, our study only proved that 0.2 mg/L BRs solution can increase the content of saikosaponin in *B. chinense*, but it is still slightly insufficient to explain the molecular mechanism of how BRs regulate saikosaponin synthesis, therefore, it is very interesting to further explore its molecular mechanism.

## Data Availability

The original contributions presented in the study are included in the article/**Supplementary Material**. Further inquiries can be directed to the corresponding author.
