# Supplementary material for: Suitable concentrations of brassinolide application enhances growth performance and saikosaponin biosynthesis by transcriptional activation of triterpenoid pathway genes in Bupleurum chinense DC
Source: Front Plant Sci. 2025 Mar 31;16:1517434. doi: 10.3389/fpls.2025.1517434 (PMC11994733; doi:10.3389/fpls.2025.1517434)
Supplement: Supplementary file 1 [file Table1.docx]

Supplementary Material

# Supplementary Figures and Tables


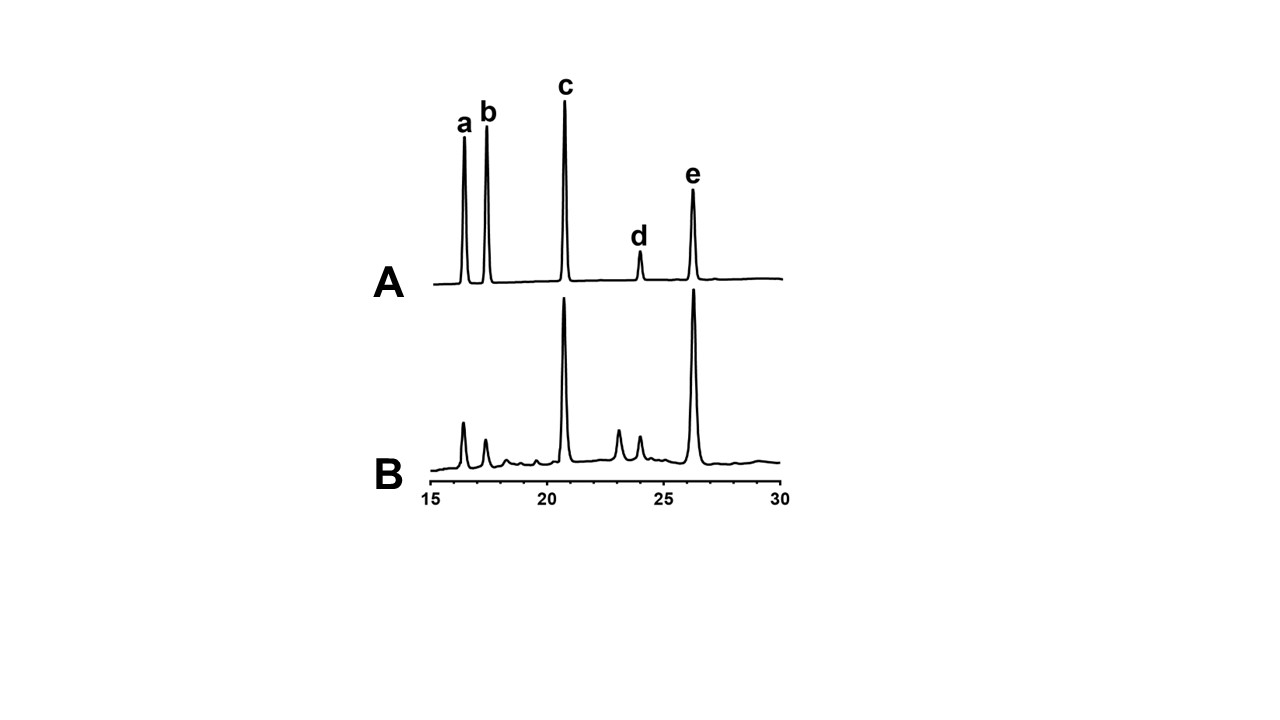


Figure S1. A: HPLC chromatogram of standard product; B: HPLC chromatogram of sample;a: saikosaponin C; b: saikosaponin F; c: saikosaponin A; d: saikosaponin E; e: saikosaponin D


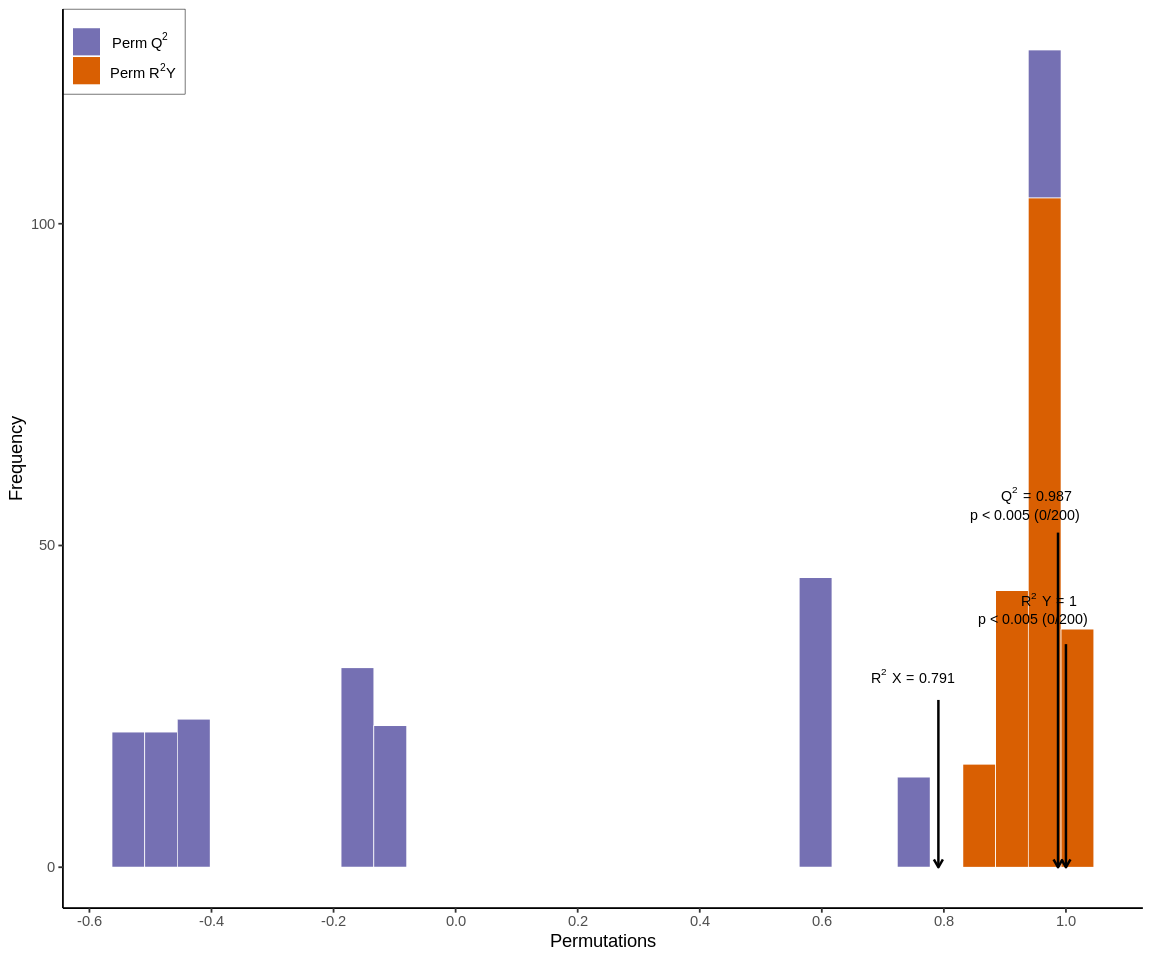


Figure S2. OPLS-DA validation plot. The horizontal axis represents the model R^2^Y, Q^2^ values, and the vertical axis is the frequency of the model classification effect in 200 random permutation and combination experiments. In the figure, orange represents the random grouping model R2Y, purple represents the random grouping model Q^2^, and the black arrow represents the R^2^X, R^2^Y and Q^2^ values ​​of the original model.

Table S3 Primer sequences of genes

| Genes | Forward primers (5'-3') | Reverse primers (5'-3') |
| --- | --- | --- |
| *AACT* | ACCCCATCAACACTAAACGCGC | CGTGCAGGTTTGCGTTTCCAGC |
| *HMGR* | TATAACTGGCGATGTGGTGAAG | GTGGCTATGAAGATTGCAGAAAC |
| *DXS* | GGGTGTGTGTGTGTGTGTGATGG | CCAAGCACAGACCTGGACTCTGG |
| *DXR* | GACGGCTGCTGTTGATCCTGG | GCAGCAAGTGCCACAACTCTGAA |
| *IPPI* | AGGTGACATTCCCTTTGGTG | AGAAGCTTCCTCTGTGCAGC |
| *FPS* | CTCACACACGCAGAGGTCAA | TGGGGATATGGTTGCGAAG |
| *SS* | GATCCTGCCAATTTCCG | CACGCCTCATTTTCACTACTC |
| *SE* | ACCTAACAAAACAGGACGGC | CATACGGATGTAGCAGGAAGAA |
| *β-AS* | ATGGTATGGGAACTGGGGTGT | ACGCCTTTACGAATGCTTTGA |
| *P450-1* | AGCATGGCCCCTGGTTGG | GCCTGCAGCCTTGGCTGATTG |
| *P450-2* | TTGGCGCAGACAGTGTGCTC | TCCAAGAGCTAAGCGCATCAGAGG |
| *P450-3* | GCAGGCGTTTACGGCCCAA | ATTGTTCCCGCGACGGTCG |
| *P450-7* | GGCATTTTGGTGTTGAGACG | TCTACAGAATCGGCCTTGGA |
| *UGT8* | GCGACATCCAAGCACCG | GCGAACAATCTCCTCCCACT |
| *UGT7382* | TTGGGTGGGGGCTTGCTAATA | CACTGCTATGTGATTGAGAAC |
| *EF-1α* | GAAGGTGACAACATGATTGAGAGG | TGAAGAGGGAGACGAAGAGGTT |
